# Rapid adaptation through genomic and epigenomic responses following translocations in an endangered salmonid

**DOI:** 10.1111/eva.13267

**Published:** 2021-07-06

**Authors:** Marco Crotti, Elizabeth Yohannes, Ian J. Winfield, Alex A. Lyle, Colin E. Adams, Kathryn R. Elmer

**Affiliations:** ^1^ Institute of Biodiversity Animal Health & Comparative Medicine College of Medical, Veterinary & Life Sciences University of Glasgow Glasgow UK; ^2^ Limnological Institute University of Konstanz Konstanz Germany; ^3^ Lake Ecosystems Group UK Centre for Ecology & Hydrology Lancaster Environment Centre Bailrigg, Lancaster UK; ^4^ Scottish Centre for Ecology and the Natural Environment University of Glasgow Rowardennan UK; ^5^ Present address: Max‐Planck Institute of Animal Behavior Am Obstberg 1 D‐78315 Radolfzell Germany; ^6^ Present address: University of Konstanz Konstanz Germany

**Keywords:** conservation translocation, DNA methylation, epiRADseq, European whitefish, geometric morphometric

## Abstract

Identifying the molecular mechanisms facilitating adaptation to new environments is a key question in evolutionary biology, especially in the face of current rapid and human‐induced changes. Translocations have become an important tool for species conservation, but the attendant small population sizes and new ecological pressures might affect phenotypic and genotypic variation and trajectories dramatically and in unknown ways. In Scotland, the European whitefish (*Coregonus lavaretus*) is native to only two lakes and vulnerable to extirpation. Six new refuge populations were established over the last 30 years as a conservation measure. In this study, we examined whether there is a predictable ecological and evolutionary response of these fishes to translocation. We found eco‐morphological differences, as functional traits relating to body shape differed between source and refuge populations. Dual isotopic analyses suggested some ecological release, with the diets in refuge populations being more diverse than in source populations. Analyses of up to 9117 genome‐mapped SNPs showed that refuge populations had reduced genetic diversity and elevated inbreeding and relatedness relative to source populations, though genomic differentiation was low (*F*_ST_ = 0.002–0.030). We identified 14 genomic SNPs that showed shared signals of a selective response to translocations, including some located near or within genes involved in the immune system, nervous system and hepatic functions. Analysis of up to 120,897 epigenomic loci identified a component of consistent differential methylation between source and refuge populations. We found that epigenomic variation and genomic variation were associated with morphological variation, but we were not able to infer an effect of population age because the patterns were also linked with the methodology of the translocations. These results show that conservation‐driven translocations affect evolutionary potential by impacting eco‐morphological, genomic and epigenomic components of diversity, shedding light on acclimation and adaptation process in these contexts.

## 1 INTRODUCTION

Conservation‐driven translocations are the intentional, human‐mediated movement and release of an organism outside its recorded range, with the aim of establishing new populations to mitigate against the extinction of important conservation units (IUCN & SCC, 2013). Predicted habitat alteration due to climate change, expansion of human activities and the introduction of invasive species are major factors prompting the use of conservation translocations to preserve biodiversity (Butchart et al., 2010; Hoegh‐Guldberg et al., 2008; Ricketts & Imhoff, 2003). Translocations have been shown to improve species conservation status (Hoffmann et al., 2010) and are projected to substantially increase as a conservation measure in future years (Swan et al., 2018).

Population‐level consequences of translocations are expected but the ecological and evolutionary responses poorly understood. Conservation translocations usually consist of small founding population sizes, which can result in failure to capture the genetic diversity of the source population and lead to a loss of genetic diversity and inbreeding (Frankham et al., 2002; Furlan et al., 2020; Jamieson, 2011). Founder effects can also lead to rapid phenotypic shifts, especially when refuge populations are introduced in areas geographically isolated from the source with no possibility of gene flow (Sendell‐Price et al., 2020). Additionally, refuge populations experience differential selection due to novel environmental pressures and in some cases have shown rapid genomic adaptation within the first few generations of a translocation (Laurentino et al., 2020; Marques et al., 2018). Unlike natural range expansions or new colonizations by dispersing individuals, the human influence on conservation translocations and the already at‐risk status of the populations are expected to have genomic consequences on the evolutionary trajectories that are difficult to predict.

Nevertheless, on short time scales there may be a lag in the evolutionary genomic responses of introduced populations due to factors such as small population sizes, time required for mutations to occur and time to linkage disequilibrium break down (Reznick et al., 2019). Epigenetics, on the other hand, provides an alternative and faster route to adaptation (Stajic et al., 2019). Epigenetic states, such as variable DNA methylation levels, change more rapidly than genetic sequence (van der Graaf et al., 2015), represent a measurable molecular marker and can change in many individuals of a population simultaneously (Angers et al., 2020). Regardless of whether this is a transient effect, transgenerational or short‐term heritability, it is suggested that epigenomic responses might facilitate population persistence and adaptation to changing environments through phenotypic plasticity and acclimation (Angers et al., 2020; Dimond & Roberts, 2020; Hu & Barrett, 2017).

A growing body of evidence from fishes in particular has shown how exposure to different environmental pressures can affect DNA methylation (Smith et al., 2015; Le Luyer et al., 2017; Gavery et al., 2018) and this contributes to the expression of phenotypic variation across different environments (Artemov et al., 2017; Campos et al., 2013; Smith et al., 2015). Furthermore, studies have found variation in DNA methylation to exceed that of standing genetic variation in some cases, suggesting a potential compensating role of epigenetics (Richards et al., 2012; Schrey et al., 2012) as an alternative route to generating phenotypic plasticity and variation (Angers et al., 2020). The epigenomic responses of natural populations to conservation translocations have rarely been explored but may provide important insight to key early stages of refuge population establishment.

**FIGURE 1:**
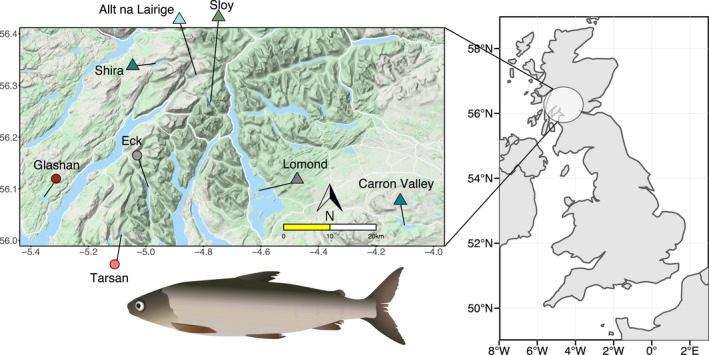
Map indicating the location of the source and refuge populations of European whitefish in Scotland, with a simplified representative fish shown. Populations from the Eck system are represented by circles, and populations from the Lomond system are represented by triangles. Source populations are in grey and refuge populations are in colour

Here, we aimed to determine consistent response to translocations at the morphological and molecular level in refuge populations of European whitefish, *Coregonus lavaretus*. In Scotland, the European whitefish (also known as powan) has a native range restricted to only two lakes, Loch Lomond and Loch Eck. These populations were colonized postglacially and are genetically closely related relative to other British populations (Crotti et al., 2020; 2021). Due to concerns for the future of these Scottish populations (Maitland & Lyle, 2013), a series of translocations were carried out over thirty years (Adams et al., 2014) (Figure 1). Between 1988 and 1990, individuals from Loch Lomond were used to establish refuge populations in Loch Sloy and Carron Valley Reservoir. Between 2009 and 2010, fish from Loch Lomond, augmented with a few individuals from Loch Sloy, were used to establish refuge populations in Lochan Shira and Allt na Lairige. Between 2010 and 2011, individuals from Loch Eck were used to establish refuge populations in Loch Tarsan and Loch Glashan. The first refuge populations (30 years before this study) were established with a much smaller number of families and released individuals compared with the later translocations (7–9 years before this study) (Adams et al., 2014; Maitland & Lyle, 2013) (see Table S1 for detailed information on the number of families, eggs, fry and adult fish released in each refuge lake). Morphological and some neutral population genetic divergence at microsatellite loci was found between Loch Lomond and the first two translocations (Etheridge et al., 2010; Præbel et al., 2021), suggesting an effect of translocation on evolutionary trajectories that could be concerning for conservation management. The full set of translocations have never been characterized for eco‐morphological, genomic or epigenomic associations with these population establishments in new environments.

Here, we used the repeated and independent translocations of whitefish populations across a time series to explore the ecological and evolutionary consequences. Repeated translocations from the same source populations provide a rare opportunity to evaluate replication in these processes and also have the potential to inform the management of future translocations (Furlan et al., 2020). Using a combined approach based on ecological, morphological, genomic and epigenomic analyses, within and across the two source and multiple refuge populations, we: (a) quantified phenotypic and ecological trait divergence and convergence; (b) assessed genome‐wide diversity and differentiation; (c) investigated differential genomic responses to selection; and (d) investigated parallel response in genome‐wide differential DNA methylation levels. Our primary focus was between source and refuge populations with the aim of inferring shared population‐level responses to the conservation measure. Collectively, these analyses provided a comprehensive insight into the molecular, ecological and evolutionary effects of human‐mediated translocations.

## 2 MATERIALS AND METHODS

### 2.1 Sample collections

European whitefish individuals were collected from eight Scottish lochs (Figure 1) in two lake translocation systems: Eck (*n* = 12 individuals; source), Glashan (*n* = 34; refuge), Tarsan (*n* = 33; refuge), which form the Eck translocation system, and Lomond (*n* = 8; source), Allt na Lairige (*n* = 9; refuge), Shira (*n* = 17; refuge), Carron Valley Reservoir (*n* = 18; refuge), Sloy (*n* = 17; refuge), which form the Lomond translocation system. Sampling occurred between August and October 2017 using multi‐panel, Nordic‐pattern gillnets. Fish collection was undertaken under licence from Scottish Natural Heritage (now NatureScot) and Marine Scotland. Individuals were photographed on the left side. White muscle tissue from the left side, underneath the dorsal fin and above the lateral line, was taken for genomic and epigenomic analyses and stored in absolute ethanol at −20°C. For stable isotope analysis (SIA), we collected ~1 cm^3^ of muscle tissue from the right side of the fish, underneath the dorsal fin and above the lateral line, and the stomach contents, and both were stored at −20°C. Due to different sampling schemes, we could not collect stable isotope data from the source population of Eck.

In addition to the samples collected in 2017, we included previously collected samples in the genomic and morphometrics analyses when available. For the genomic analyses, we included a subset of the parent fish from Loch Lomond (*n* = 40), Loch Sloy (*n* = 17) and Loch Eck (*n* = 41) that were used to establish the refuge populations between 2009 and 2011. For the morphometric analyses, we added photographs from: a sampling of the parent fish from Loch Lomond (*n* = 89) and Loch Eck (*n* = 118) that were used to establish the refuge populations between 2009 and 2011; samples from refuge populations Allt na Lairige (*n* = 4), Lochan Shira (*n* = 15), Loch Glashan (*n* = 33) and Loch Tarsan (*n* = 60) collected during a survey in 2014 and 2015 (Lyle et al., 2017); and samples from Loch Lomond (*n* = 21), Carron Valley Reservoir (*n* = 11) and Loch Sloy (*n* = 20) collected in a survey in January 2018.

### 2.2 Geometric morphometric analysis

All photographs used for morphometric analyses were taken with the same protocol and using graph paper or ruler for scale. Body shape was captured with 14 fixed landmarks (Figure S1a) chosen based on previous studies and for their established functional importance in foraging and locomotion (Jacobs et al., 2019; Siwertsson et al., 2013) (*N* = 508 individuals). Landmarks were digitized using *TPSDig2* v.2.16 (Rohlf, 2010). Statistical analyses were conducted in the R environment (R Core Team, 2019) with the package *geomorph* v.3.0.7 (Adams and Otárola‐Castillo, 2013). A Generalized Procrustes Analysis was performed to remove variation due to size and orientation of individuals. We tested for homogeneity of allometric curves using the function *procD*.*allometry*. The linear model used was *Shape* *~log(Size)* * Lake, with *Shape* being the combination of all principal components, and *Size* the centroid size (the square root of summed squared distances of landmarks from the configuration centroid). We implemented the *procD*.*allometry* function for each lake system separately. When the interaction term was significant, we performed a pairwise test for homogeneity of slopes using the *advanced*.*procD*.*lm* function, to test if populations differed in allometric slope. If the interaction term was not significant, that is if different populations have common or parallel trajectories, we performed pairwise tests for shape difference. Significance was assessed with a randomized residual permutation procedure with 1000 iterations. We performed a principal component analysis (PCA) on the Procrustes coordinates of all individuals to explore the major axes of variation.

We performed a phenotypic trajectory analysis (PTA) (Collyer & Adams, 2013) in *geomorph* to quantify the level of parallelism, or deviation from it, in body shape change in response to the translocations across the two lake systems. Significant difference in trajectory direction (*θ*_P_: differences in the direction of phenotypic change) and trajectory lengths (ΔL_P_: differences in the magnitude of phenotypic change) was assessed using 1000 permutations.

### 2.3 Linear trait analysis

Linear measurements of nine body traits plus fork length were obtained from distance between landmarks (Figure S1b) (*N* = 508 individuals). Traits were chosen based on previous publications (Jacobs et al., 2019; Siwertsson et al., 2013) to represent functionally relevant features that respond to differences in diet and environment. Because these linear traits are correlated to fish body length, they were first corrected for allometry following Siwertsson et al. (2013). Briefly, to reduce variance each trait was log10‐transformed; then, we calculated a common slope for each trait using an ANCOVA with the formula *Trait* *~Lake* * Size. The slope was then used to in the formula (Siwertsson et al., 2013):

$$\log_{10}Y_{\text{st}}=\log_{10}Y_{\text{obs}}+b\left(\log_{10}L_{\text{st}}‐\log_{10}L_{\text{obs}}\right)$$

where Y_st_ is the standardized trait value, Y_obs_ is the observed trait value, *b* is the slope of the ANCOVA, L_st_ is the average length of all whitefish examined, and L_obs_ is the measured body length of each fish. Divergence in linear traits between lake systems and lakes was then compared using a Kruskal–Wallis test with a *post hoc* Dunn test with the Benjamini–Hochberg (BH) correction for multiple testing. A PCA was carried out to determine the major axes of phenotypic variation between source and refuge populations, across and within lakes.

### 2.4 Stable isotope analysis

Lipid extraction of tissue and stable isotope measurement methods followed Yohannes et al. (2017). Isotopic turnover rate of muscle tissue reflects diet during the preceding 2–4 months (Vander Zanden et al., 2015), while stable isotope of stomach content reflects very recent diet; by using these two values we could compare how stable diet is in these populations. Muscle tissue was dried in an oven at 50°C for 48 hours. Briefly, the dried muscle (Glashan = 10, Tarsan = 10, Lomond = 8, Allt na Lairige = 9, Shira = 10, Carron Valley = 9, Sloy = 10) and stomach content (Glashan = 10, Tarsan = 9, Lomond = 6, Allt na Lairige = 8, Shira = 9, Carron Valley = 10, Sloy = 3) samples were immersed in a 2:1 chloroform:methanol solvent with a volume four times that of the sample. Samples were mixed for 30 s, rested for 20 min, centrifuged for 5 min at 1295 *g* and the supernatant was removed. We repeated this process three to four times, until the supernatant was clear. Samples were then rinsed in distilled water and dried at 60°C for 48 h. Sub‐samples of 0.7–1 mg were combusted in a vario Micro cube elemental analyser (Elementar, Analysensysteme). Stable isotope ratios of carbon (^13^C/^12^C) and nitrogen (^15^N/^14^/N) were measured with an IsoPrime (Micromass, Manchester, UK) isotope ratio mass spectrometer.

Stable isotope analysis was conducted using the framework proposed by Cucherousset and Villegér (2015), using the *si_div*.*R* set of functions (Cucherousset & Villegér, 2015) in R (R Core Team, 2019). For each population, we first calculated isotopic richness IRic and isotopic divergence IDiv. Isotopic richness represents the total extent of multidimensional foraging niche space used by populations, that is the convex hull area, while isotopic divergence quantifies the distribution of populations within isotopic space, with values of 0 indicating populations are close to the centre of gravity and of 1 when close to the edge of the convex hull. The analyses were run on scaled, unitless (zero to one) coordinates (Cucherousset & Villéger, 2015).

### 2.5 EpiRADseq and ddRADseq library preparation

Samples collected in 2017 were prepared using epiRADseq (Schield et al., 2016) for genomic and epigenomic analyses (Table 1). Genomic SNPs from ddRADseq and epiRADseq are equivalent for estimating genetic diversity and population structure (Crotti et al., 2020). The parent fish were prepared using ddRADseq (Peterson et al., 2012) for genomic analyses only, because only their fin tissue was available and DNA methylation is tissue‐specific. The protocol used for the ddRADseq and epiRADseq libraries follows Jacobs et al. (2019), with minor modifications described in Crotti et al. (2020). The ddRADseq libraries used *PstI*‐HF and *MspI* enzymes (New England Biolabs) and the epiRADseq libraries used *PstI*‐HF and the methylation‐sensitive *HpaII* enzymes. The enzymes *MspI* and *HpaII* have the same recognition site. Libraries were sequenced on an Illumina NextSeq500 with 75‐bp paired‐end reads at Glasgow Polyomics to a depth of 400 M reads each.

**TABLE 1:** List of populations sampled, the lake system they belong to, whether they are source or refuge, the year refuge populations were established and the life stage of translocated individuals, expected (*H*_E_) and observed (*H*_O_) heterozygosity and nucleotide diversity (π)

Lake	System	Lake type	Year of refuge translocation	Life stage introduced	*H*_E_	*H*_O_	π
Eck	Eck	Source			0.345	0.361	0.00378
Glashan	Eck	Refuge	2010–2011	Fry, adults	0.331^*^	0.352^*^	0.00369
Tarsan	Eck	Refuge	2010–2011	Fry, adults	0.334^*^	0.350^*^	0.00374
Lomond	Lomond	Source			0.329	0.351	0.00366
Allt na Lairige	Lomond	Refuge	2009–2010	Eggs, fry	0.305^*^	0.335^*^	0.00359^*^
Shira	Lomond	Refuge	2009–2010	Eggs, fry	0.319^*^	0.337^*^	0.00365
Carron Valley Reservoir	Lomond	Refuge	1988–1990	Fry	0.308^*^	0.327^*^	0.00351^*^
Sloy	Lomond	Refuge	1988–1990	Fry, adults	0.309^*^	0.326^*^	0.00347^*^

* Significant differences in heterozygosity and nucleotide diversity in refuge populations from the source.

The epiRADseq data set was composed of 113 individuals (1.3–15.1 M reads per sample) split among three libraries, and the ddRADseq library was composed of 96 individuals (2.5–9.1 M reads per sample) (Table S2).

### 2.6 Genotyping‐by‐sequencing data processing

First, raw reads were demultiplexed with *process_radtags* in *Stacks* v.2.4.1 (Catchen et al., 2013; Rochette et al., 2019) and trimmed to 65 bp, and both forward and reverse reads were retained. We then trimmed the first 5 and 3 bp with *Trimmomatic* (Bolger et al., 2014) from the forward and reverse reads to remove the enzyme cut site, and paired‐end trimming was done with the following settings: LEADING = 20, TRAILING = 20, to remove low‐quality reads, and CROP = 60, so that reads were all of the same length. As a reference genome, we used a chromosome‐level assembly (GCA_902810595.1) of *Coregonus sp*. ‘Balchen’ (De‐Kayne et al., 2020) which is part of the Alpine lineage of the same European whitefish species complex as the Scottish samples, and split from the Scottish lineage before the last glacial maximum *ca*. 21 K years ago (Hudson et al., 2011; Crotti et al., 2021). Reads were mapped to the genome using *bwa mem* v.0.7.17 (Li & Durbin, 2009) with default settings and retained if mapping quality was >20 with *samtools* v.1.7 (Li et al., 2009). After mapping to the reference genome, samples retained on average 4.1 M reads (SD = 1.9 M). We assembled loci using *Stacks* v.2.4.1 and the *ref_map*.*pl* script. Genotyping in *Stacks* resulted in a total of 1,234,536 loci, with an average effective per‐sample coverage of 11.6x (SD = 4.3x, min = 4.3x, max = 29.9x). A principal component analysis (PCA) revealed the presence of a batch effect between the epiRADseq and ddRADseq libraries on PC2. To identify and exclude the loci responsible, we used two approaches: (a) we ran a PCA and calculated the correlation between eigenvectors and SNP genotype using the *snpgdsPCACorr* function in the R package *SNPRelate* v. 1.16 (Zheng et al., 2012), and (b) we ran a PCA and calculated the loading factor for each SNP on PC2 in the R package *adegenet* v. 2.1.1 (Jombart, 2008). Loci for which SNPs showed a correlation or loading factor higher than 0.3 were considered as strongly correlated with library type (Ratner, 2009) and added to a blacklist (total number of blacklisted loci =737) in *Stacks* and excluded from further analyses.

### 2.7 Genotyping and filtering for genomic analyses

We generated three data sets for population genomic analyses: a combined data set with all eight populations specifically for outlier analyses, and a data set for the Eck system (i.e. Eck, Glashan and Tarsan) and for the Lomond system (i.e. Lomond, Allt na Lairige, Shira, Carron Valley Reservoir, Sloy) separately that were used for the genetic diversity, inbreeding and relatedness analyses. An initial vcf file was generated for each in *populations* (part of the *Stacks* pipeline), with the following criteria: ‐p 6 (minimum number of populations genotyped), ‐r 0.75 (minimum proportion of individuals genotyped per population), ‐‐min‐maf 0.05 (global minor allele frequency filter), ‐‐max‐obs‐het 0.6 (maximum observed heterozygosity required to process a site at a locus) for the combined data set; ‐p 4, ‐r 0.75, ‐‐min‐maf 0.05, ‐‐max‐obs‐het 0.6 for the Lomond system data set; and ‐p 2, ‐r 0.75, ‐‐min‐maf 0.05, ‐‐max‐obs‐het 0.6 for the Eck system data set. One SNP per locus was retained.

Each data set was then filtered in *vcftools* v.0.1.15 (Danecek et al., 2011), retaining SNPs that fulfilled the following criteria: a minimum sequencing depth of 5 per individual, a minimum mean sequencing depth of 8 across individuals, a maximum mean sequencing depth across individuals of 40 (to remove possible repetitive reads), a minor allele frequency (MAF) of 0.05 and a 33% missing data threshold. After this step, we excluded individuals with more than 30% missing genotypes. We then removed SNPs out of Hardy–Weinberg equilibrium (HWE) within populations using the script *filter_hwe_by_pop*.*pl* (available at https://github.com/jpuritz/dDocent/blob/master/scripts/filter_hwe_by_pop.pl) and with the script *pop_missing_filter*.*sh* (available at https://github.com/jpuritz/dDocent/blob/master/scripts/pop_missing_filter.sh) removed sites with more than 33% missing data per population. After filtering, the genomic combined data set comprised 184 individuals and 5116 SNPs, the Lomond system data set comprised 110 individuals and 6333 SNPs, and the Eck system data set comprised 77 individuals and 3712 SNPs.

Prior to the redundancy analysis, the combined data set was split between populations from the Lomond and Eck system for missing data imputation. We imputed missing data using the *LD*‐*kNNi* method implemented in *Tassel* v.5 (Bradbury et al., 2007), based on the 10 closest genotypes using the default settings, and re‐merged into a combined data set using *bcftools* v.1.8.

### 2.8 Genotyping quality assessment

To assess the quality of the ddRADseq and epiRADseq data for combined genomic analyses we: (a) calculated the heterozygous miscall rate, which measures putative genotyping errors by estimating deviation from HWE, with the R package *radiator* (Gosselin, 2020) and (b) calculated rarefied allelic richness with the R package *hierfstat* v.0.04‐22, down‐sampling to eight samples per population (Goudet, 2005), using epiRADseq and ddRADseq samples separately for each lake sample that had both epiRADseq and ddRADseq samples (Lomond, Sloy, and Eck). The aim of these tests was to identify any deviations between data sets that would be indicative of genotyping errors, which it would influence downstream analyses. In addition, we estimated the genotyping error rate due to low sequencing coverage in the combined population genomics data set with the *ErrorCount*.*sh* script (https://github.com/jpuritz/dDocent/blob/master/scripts/ErrorCount.sh).

### 2.9 Genetic diversity, relatedness, inbreeding and differentiation

Summary statistics of genetic diversity (expected heterozygosity H_E_, observed heterozygosity *H*_O_), nucleotide diversity π and number of private alleles per population were calculated by the *population* module of *Stacks* for each lake system separately. For these analyses, we retained all SNPs present in the loci from the Lomond (9117 SNPs in total) and Eck (5249 SNPs in total) system data sets, as these metrics do not need to account for linkage disequilibrium. Genomic measures of pairwise relatedness, *R_xy_*, and individual inbreeding coefficient, *F_H_*, were estimated in *Plink* v.1.9 (Chang et al., 2015; Purcell et al., 2007) with the *make*‐*rel* and *het* functions, respectively, following Waters et al. (2020). Unbiased estimates of inbreeding rely on allele frequencies being derived from an outbred population of unrelated individuals, and with SNPs in linkage equilibrium (Kardos et al., 2015). Therefore, we further filtered the genomic Lomond and Eck system data sets in *Plink*, retaining SNPs with *r* ^2^ < 0.2 within 1 Mb windows, and with a MAF of 0.05 in the source populations, retaining 3553 in the Lomond system and 2083 SNPs in the Eck system genomic data sets, respectively. *R_xy_* measures the expected proportion of shared alleles between individual pairs that are identical by descent, while *F_H_* compares the observed number of homozygous genotypes to the expected mean number under random mating (Taylor, 2015). Differences in the distribution of pairwise relatedness and inbreeding coefficient between populations were tested with Kolmogorov–Smirnov tests in R (R Core Team, 2019), as the data were not normally distributed.

To gain an insight into the impact of founder size on genetic diversity, inbreeding and relatedness in refuge populations, we regressed the number of families used to create the refuge populations (Table S1) against the average decrease in observed heterozygosity (in percentage) and average increase in inbreeding and relatedness in the refuge populations in R.

Between population Weir and Cockerham *F*_ST_ (Weir & Cockerham, 1984) was calculated in *GenoDive* (Meirmans & Van Tienderenn, 2004) and significance assessed with 10,000 permutations. We employed a maximum‐likelihood approach for population assignment with *Admixture* v.1.3.0 (Alexander et al., 2009). We ran analyses with a 20‐fold cross‐validation (CV), and tested *K* values ranging 1–5, and the optimal value was defined as the one with the lowest CV error. Furthermore, we ran a principal component analysis with *SNPRelate* for all the three data sets. The pairwise *F*_ST_ and *Admixture* analyses were run on each lake system data set separately.

### 2.10 Detection of outliers

To detect genomic outlier SNPs associated with translocations, we used two approaches. First, we applied a redundancy analysis (RDA) to the combined data set as a multilocus genotype–environment association (GEA) using the R package *vegan* v.2.5‐3 (Oksanen et al., 2018). RDA is a multivariate approach that can simultaneously analyse the response of thousands of genomic variants to predictors of choice and is thus suitable for genotype–environment association (Forester et al., 2018). Briefly, RDA uses constrained ordination to model a set of explanatory variables and unconstrained ordination axes to model the dependent variables (Forester et al., 2018). SNPs that load heavily on one or more explanatory variables are considered outliers. The dependent variable was the multilocus genotype (each genomic SNP), and the explanatory variables were the lake type (source or refuge) and lake system (source population Lomond or Eck). Significance of the RDA was assessed by performing an analysis of variance (ANOVA) with 1,000 permutations. The percentage of variation explained by the RDA (R^2^) was calculated using the function *RsquareAdj* in *vegan*. SNPs with a loading greater than ±2.5 standard deviation, or *z*‐score, (equivalent to a two‐tailed *p*‐value = 0.01) on the lake type RDA axis were considered to be outliers.

Second, we used a Bayesian framework implemented in *BayPass* (Gautier, 2015). As with the RDA analysis, we looked for an association between genotype and lake type (source or refuge) as a binary covariate using the AUX model. *BayPass* accounts for confounding demographic effects by estimating a covariance matrix of allele frequencies between populations, so we did not include lake system as a covariate. The AUX model uses Bayes Factors (BFs) to identify SNPs associated with covariates based on a calibration procedure using pseudo‐observed data sets (PODs; Gautier, 2015).

To visualize the location of the outlier SNPs recovered by RDA and *BayPass* across the genome, we averaged the frequency of the major allele over the two source populations and the six refuge populations, respectively, calculated the difference and the relative z‐score, and plotted the z‐score of the absolute allele frequency change per SNP.

### 2.11 EpiRADseq data processing

EpiRADseq relies on the comparison of read counts to detect loci that are differentially methylated between groups (Schield et al., 2016). To assemble loci, we mapped the quality trimmed fastq files against the genome‐referenced *Stacks* catalogue from the population genomic analysis using *bwa mem* with default settings. Read counts at each locus were extracted using the *samtools idxstats* command for all individuals separately and subsequently combined to create a count table for each lake system separately.

Preliminary analyses using multidimensional scaling (MDS) in the R package *edgeR* v.3.24.3 (Robinson et al., 2010) revealed a sequencing library batch effect. It was not possible to incorporate batch effect in the model because individuals from the eight populations were not represented equally across the three epiRADseq libraries. Therefore, we subsetted the count table to contain only the populations for which individuals were spread across the different libraries and used a negative binomial generalized linear model with the function *glmFit* in *edgeR* to identify loci for which read counts were influenced by library. Loci with false discovery rate (FDR) <0.05 were then excluded from the count table. After removing the library effect, we identified a weak batch effect associated with the Illumina adapter barcode. We reiterated the same procedure and excluded loci affected by this bias. Finally, we excluded loci that had nonzero read counts in fewer than 33% of individuals from each lake system separately to remove uninformative loci present in only a small number of individuals. After filtering, the epigenomic Lomond system (*N* = 66 individuals), Eck system (*N* = 45) and combined data sets (*N* = 111) had totals of 120,897, 117,395 and 114,565 loci, respectively.

### 2.12 Differential DNA methylation analysis

Differential methylation patterns between source and each refuge population were examined for the Lomond and Eck system separately using the *glmFit* and *glmLRT* functions in *edgeR*. All loci with an FDR < 0.05 for each comparison were considered to be differentially methylated (DM). Excess of DM loci sharing between source‐refuge population comparisons was calculated with the R package *SuperExactTest* v.1.0.7 (Wang et al., 2015).

To explore the major axes of epigenomic variation shared across groups, we log‐transformed the read counts with the function *rld* and performed a PCA in *pcaMethods* v.1.74 (Stacklies et al., 2007) for the combined data set and each lake system separately. Additionally, to identify loci with methylation levels associated with lake type (source or refuge), we conducted an RDA on the log‐transformed read counts of the combined read count table (dependent variable), using lake type and lake system as explanatory variables (as in the genomic RDA). Loci with z‐transformed loading greater than ±2.5 on the lake type RDA axis were considered to be outliers. Because DNA methylation levels are also influenced by the age of the individual (Angers et al., 2020), we ran a separate RDA with the addition of age as explanatory variable, to assess whether the observed epigenomic patterns were driven by this variable.

### 2.13 Gene ontology analyses

To explore putative functions, we analysed outlier SNPs and DM loci using gene ontology (GO) annotations. Genes overlapping the SNPs and DM loci and genes within 3000 bp upstream and downstream of these outliers were retained for the analysis. Protein sequences from the European whitefish genome (De‐Kayne et al., 2020) were mapped and annotated to the SwissProt database using *Blast2Go* (Götz et al., 2008). Loci were annotated with *BEDTools* v.2.27.1 (Quinlan & Hall, 2010) to identify genes and associated proteins from the European whitefish genome. Over‐representation tests were conducted in *PANTHER* (Mi et al., 2009; Thomas et al., 2003) using Fisher's exact test. Genes were considered as significantly enriched if FDR < 0.1. The set of genes overlapping the STACKS loci was used as background data set for the GO enrichment analysis.

### 2.14 Genomic and epigenomic association with morphological variation

We aimed to disentangle the association of genomic and epigenomic with morphological variance following translocation, across lake system and age since establishment. To do so, we conducted one RDA and two partial RDAs (pRDAs) to partition the percentage of morphological variation due to genomic and epigenomic effects together, *morpho* *~gen* + *epi*, proportion of morphological variation explained by genomic effects while accounting for epigenomic variation, *morpho* *~gen* + *Condition(epi)*, and proportion of morphological variation explained by epigenomic effects while accounting for genomic variation, *morpho* *~epi* + *Condition(gen)*, following a similar approach by Rougeux, Laporte, Gagnaire, & Bernatchez (2019). We ran these analyses using the first three PCs from the morphological PCA on body shape (comprising 54% of total variation), the PCA on the genomic combined data set and the PCA on the epigenomic combined data set, using all lakes and each lake system separately. We included only the first three PCs from both the genomic and epigenomic PCAs as proportion of variation explained declines rapidly after PC1 in both analyses (Figure S6). Estimation of morphological variance explained by genomic‐epigenomic interactive effect was computed with the function *varpart* in *vegan*. Because DNA methylation variation arises more rapidly than genetic variation (van der Graaf et al., 2015), we tested if genomic and epigenomic variation correlated differently with morphological variation at different stages of population divergence. For this, the Lomond system was split into two groups, source and young (7–9 years old) refuge populations, and source and old (30 years old) refuge populations.

## 3 RESULTS

### 3.1 Morphological analyses

Morphological analyses revealed a combination of lake‐specific patterns and some general trends of similarity across refuge populations. Testing homogeneity of allometric slopes showed a significant association between body shape and the interaction between body size and lake of origin for the Eck system (*F*_2,277_ = 5.72, *p*‐value = 0.001), with the refuge populations having different allometric trajectories compared with the source population (Table S3). For the Lomond system, we found a significant interaction between body size and lake when all populations were included in the model (*F*_4,215_ = 2.2, *p*‐value = 0.01), but not when Shira was excluded (*F*_3,191_ = 0.85, *p*‐value = 0.405), indicating that Shira fish had a different allometric trajectory compared with the other populations (Table S3). All other refuge populations from the Lomond system showed similar allometric trajectory but significant body shape differences compared with the source (Table S3).

**FIGURE 2:**
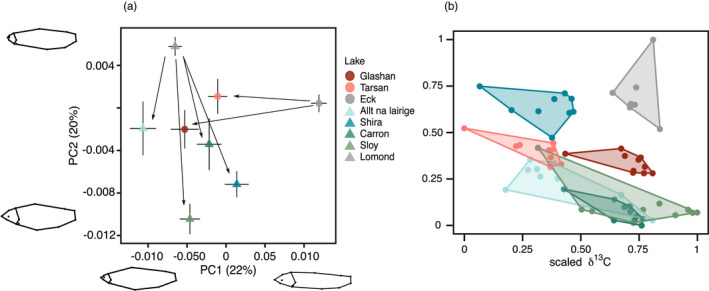
Morphological and stable isotope analyses. (a) Principal component analysis for all populations. Arrows indicate direction of body shape change from source to refuge populations. Body shape differences between highest and lowest values of PC1 and PC2 are reported on each axis. Points represent mean value for each population, with bars showing standard error of the mean. (b) Convex hull area of the scaled stable isotopes for muscle tissue, which are described by the IRic index

The PCA of body shape showed there are differences in average body shape within and between systems (Figure 2a, Figure S2a). In the Eck system, individuals from the refuge populations had smaller heads and larger bodies compared with individuals from the source, while in the Lomond system refuge, populations had larger heads and smaller bodies compared with the source (Figure 2a). The refuge populations were grouped more closely on PC1 (22% of variation) and PC2 (20% of variation) than the source populations. The next three PCs combined explained 27% of the variation.

The phenotypic change in body shape between source and the combined refuge populations in the two lake systems was similar in magnitude, as inferred from PTA (ΔL_P_ = 0.001, *p*‐value = 0.4). However, the direction of phenotypic change between source and refuge populations differed significantly across systems (*θ*_P_ = 96.14°, *p*‐value = 0.001); the different directions resulted in a convergence of the source to refuge trajectories on PC1 (Figure S2b).

In linear traits, all populations were generally similar, with only body depth posterior, caudal peduncle length and fin length differing significantly in most source‐refuge population comparisons across lake system (Table S4, Figure S3). The Eck system refuge populations differed from the source population in more body measurements than did the Lomond system (Table S4, Figure S3).

### 3.2 Ecological niche

We found a positive relationship between δ^15^N from muscle and δ^15^N from stomach content (*F*_1,5_ = 17.03, *p*‐value < 0.01), with the Loch Lomond and Carron populations showing the highest δ^15^N levels; Glashan and Tarsan intermediate levels; and Allt na Lairige, Shira and Sloy the lowest (Figure S2c). This relationship between muscle and stomach content δ^15^N isotopes indicates that the difference in diet is maintained over time, integrated from food to muscle.

In the Lomond system, isotopic richness (IRic) and isotopic divergence (IDiv) were higher in most refuge populations compared with source (Table S5). This suggests that the diversity of diet of the source population was lower than that of the refuge populations. In the Eck system, there was little difference between the two refuge populations, with Tarsan having slightly higher IRic and IDiv (Table S5). There was no overlap in isotopic niche space between the Lomond source population and its refuge populations (Figure 2b).

### 3.3 Genotyping quality assessment

The heterozygous miscall rate was <0.1% for the ddRADseq and epiRADseq samples (Table S6). Rarefied allelic richness in each lake was nearly identical regardless of genotyping method (0%–1.0% difference; Table S7). Assuming all low depth homozygote genotypes in the genomic combined data set were errors, the estimated genotyping error rate due to low read depth was 0.03%. Thus, the genomic data were concluded to be high quality when generated from epiRAD or ddRAD libraries (consistent with Crotti et al., 2020).

### 3.4 Genetic diversity

Genetic diversity and heterozygosity were generally lower in the refuge populations than the source; the difference is very small but significant in most cases (Table 1). No refuge population had any private allelic richness while source lakes had some—though few—private alleles (14 private alleles in Eck, two private alleles in Lomond).

**FIGURE 3:**
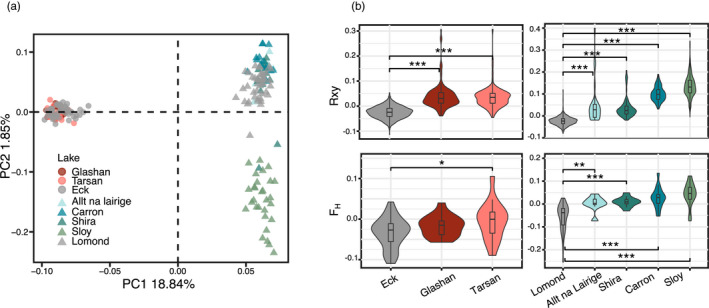
Population genomic analyses. (a) Principal component analysis of the full genomic data set displaying PC1 and PC2. (b) Distribution of pairwise relatedness (*Rxy*) and inbreeding coefficient (*F*_H_) indices for the Eck and Lomond system data sets. Significant differences were observed for all comparisons between source and refuge, except for Glashan which did not differ in *F*_H_ from the Eck population

Population inbreeding coefficients *F*_H_ were higher in the refuge populations; significantly so in all but one instance (Table 2, Figure 3b). Relatedness *R_xy_* was higher in the refuge populations than in the source (Table 2, Figure 3b). *R_xy_* in the 30‐year‐old refuge populations of the Lomond system were higher than in the 7‐ to 9‐year‐old populations (Figure 3b).

**TABLE 2:** Test statistic, *p*‐value and the direction of the significant differences from the Kolmogorov–Smirnov tests for *F*_H_ and *R*xy indices. Tests were conducted between source (S) and refuge (R) populations for the Eck and Lomond systems

Comparison	Lakes	*Rxy*			*F*_H_		
		D	*p*‐value	Difference	D	*p*‐value	Difference
Eck system	Eck vs Glashan	0.74	<0.001	R > S	0.32	0.14	NA
	Eck vs Tarsan	0.72	<0.001	R > S	0.39	0.04	R > S
Lomond system	Lomond vs Allt na Lairige	0.66	<0.001	R > S	0.69	<0.001	R > S
	Lomond vs Shira	0.82	<0.001	R > S	0.72	<0.001	R > S
	Lomond vs Carron	0.99	<0.001	R > S	0.69	<0.001	R > S
	Lomond vs Sloy	0.99	<0.001	R > S	0.82	<0.001	R > S

There was a trend that diversity might be associated with founding population size. We found that populations with greater numbers of founders had more genetic diversity (*F*_1,4_ = 89.47, *p*‐value < 0.001), and showed lower inbreeding (*F*_1,4_ = 76.9, *p*‐value < 0.001) and lower relatedness (*F*_1,4_ = 12.24, *p*‐value = 0.02) (Figure S3). This co‐varies with inbreeding being slightly higher in the two 30‐year‐old refuge populations (Sloy, Carron) relative to the 7‐ to 9‐year‐old populations (Figure 3b). Due to the translocation design being a real‐world conservation measure rather than an evolutionary experiment, we cannot tease these influences apart more robustly.

### 3.5 Genetic differentiation

The major source of population genomic variation among individuals was clearly by lake system (Eck or Lomond) (PC1 19%) (Figure 3a). Individuals from different populations within the Eck system were not genetically differentiated (i.e. a lack of separation on PC1, PC2 or [not shown] PC3), while in the Lomond system the 30‐year‐old translocated populations (Sloy, Carron) separated from the 7‐ to 9‐year‐old populations along PC2. This concurred with admixture analyses, which suggested two genetic clusters (*K* = 2) as the best‐fitting scenario; the Sloy population significantly differentiated from Lomond, and three clusters as the second best‐fitting scenario, with the Carron population further splitting from Lomond but no further genetic structuring by refuge lake (Figure S5). Admixture analysis on the Eck system data set found no structuring between the source and refuge populations (*K* = 1 as the best‐fitting scenario) (Figure S5).

Population‐level genetic differentiation was low to moderate between source and refuge populations in both systems. The Eck source population was slightly, albeit significantly, differentiated from refuge populations (*F*_ST_ = 0.002–0.003, *p*‐value < 0.05), and the two refuge populations were not differentiated from each other (*F*_ST_ = 0.0001, *p*‐value > 0.05) (Table 3). In the Lomond system, *F*_ST_ between source and refuge populations ranged up to 0.030, with all source‐refuge comparisons being significantly different (*p*‐value < 0.05). There was a trend of age effect, with differentiation being higher between the source and two 30‐year‐old refuge populations than between the source and the two 7‐ to 9‐year‐old ones, and there was no significant differentiation between the two 7–9 refuge populations (Table 3).

**TABLE 3:** Pairwise Weir and Cockerham *F*_ST_ between each population for the Eck and Lomond systems

Eck system	Eck	Glashan	Tarsan		
Eck	‐				
Glashan	**0.003**	‐			
Tarsan	**0.002**	0.0001	‐		
Lomond system	Lomond	Allt na Lairige	Shira	Carron Valley	Sloy
Lomond	‐				
Allt na Lairige	**0.006**	‐			
Shira	**0.004**	0.003	‐		
Carron Valley	**0.020**	**0.030**	**0.026**	‐	
Sloy	**0.030**	**0.041**	**0.032**	**0.050**	‐

Note

Bold values indicate significant differentiation.

### 3.6 Genomic outliers of translocation

**FIGURE 4:**
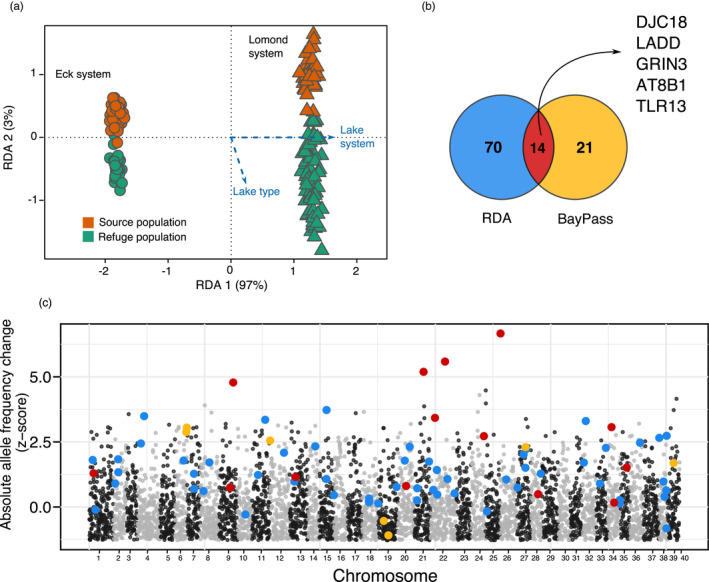
Genomic outliers of translocation. (a) Redundancy analysis (RDA) of the combined genomic data set, using lake system (97% of variation, RDA 1) and lake type (3% of variation, RDA 2) as response variables. (b) Number of outlier SNPs identified by RDA (70, blue), *BayPass* (21, yellow) and the SNPs that overlap (14, red). The gene IDs correspond to the shared outlier genes between RDA and *BayPass*. (c) Distribution of RDA (blue), *BayPass* (yellow) and shared (red) outlier SNPs along the genome. The y‐axis represents the absolute allele frequency change (z‐score) between source and refuge populations. Chromosomes are coloured alternating black and grey

We investigated genomic signals of selection due to translocation, defined as regions of the genome consistently identified as outliers between source and refuge populations across lake systems. Using both lake type (source or refuge) and lake system as explanatory variables, the genomic RDA explained 18.7% (adjusted *R* ^2^ = 0.187) of the total variance (*F*_2,184_ = 22.4, *p*‐value = 0.001). Of this, lake system explained 96.6% on axis 1, and lake type explained 3.4% (Figure 4a), separating source and refuge populations on axis 2. From the RDA, we identified 70 outlier SNPs associated with lake type (Figure 4b). The analysis implemented in *BayPass* identified 21 outlier SNPs associated with lake type, 14 of which overlapped with the outliers of the RDA analysis (Figure 4b). Forty of the 77 outlier SNPs could be mapped to genes (±3000 bp) in the whitefish reference genome (Table S8). Outlier SNPs from the two approaches were found to be distributed across the genome (Figure 4c).

From the 14 outlier SNPs shared across both approaches (RDA and BayPass), five were found in or near genes and so could be putatively associated with functions (Figure 4b, Table S8). These genes were DnaJ homolog subfamily C member 18 (DnaJC18), ladderlectin (LADD), G protein‐regulated inducer of neurite outgrowth 3 (GPRIN3), Atp8b1 and Toll‐like receptor 3 (TLR3).

### 3.7 Differential methylation

Across all lakes, 1294 loci were differentially methylated (DM loci) between source and refuge populations. Most of the variation in the data set was explained by lake system. Specifically, PC1 (5% of the total variation) separated Eck system from Lomond, and refuge populations separated from the source populations on PC3 (1.8% of the total variation) (Figure S6a,b). The DM loci were distributed mainly in intergenic regions (61%–62%) and within genes (29%–30%) (Figure S7).

**FIGURE 5:**
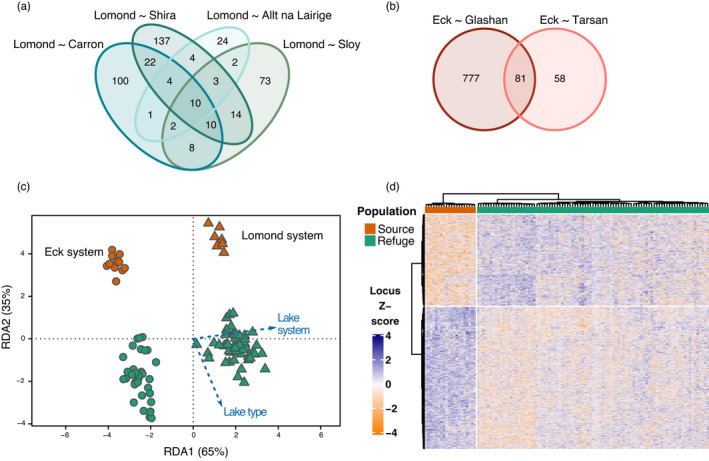
Results of the epigenomic analyses. (a) Number of differentially methylated loci between source and refuge populations in the Lomond system shared across comparisons. (b) Number of differentially methylated loci between source and refuge populations in the Eck system shared across comparisons. (c) Redundancy analysis (RDA) of the combined epigenomic data set, with lake system (65% of variation) and lake type (35% of variation) as response variables. (d) Heatmap of the normalized log_2_‐transformed 1493 loci identified by the RDA as associated with lake type. Rows represent the loci, and columns represent individuals. Locus Z‐score represents the number of standard deviations of away from the mean of the log‐transformed read counts in the data set for each sample

DM loci were unique to each lake system, with only one locus shared across systems (Locus 77123 on chromosome 3). In the Eck system, there were 139 DM loci between Eck and refuge population Tarsan, and 858 DM loci between Eck and refuge population Glashan. Of these, 81 loci overlapped, which was more than expected by chance (*p*‐value < 0.0001, Figure 5b) and 24 were found in or near genes (Table S9). In the Lomond system, there was less variation in the number of DM loci between source and refuge populations, ranging between 50 and 204 (Figure 5a). Ten DM loci were shared across all four comparisons, which was more than expected by chance (*p*‐value < 0.0001) (Figure 5a), one of which mapped within a gene (Table S9), DPYSL5.

There were no significantly enriched GO terms (FDR > 0.1) from the genes associated with DM loci (Eck system), but the top 11 GO terms (based on uncorrected *p*‐value < 0.001, fold enrichment = 8.96–90.65) included neural functions (e.g. GO:0099536, synaptic signalling) and ion transport (e.g. GO:1901380, negative regulation of potassium ion transport) (Table S10). The gene DPYSL5, which was shared across all Lomond system populations, may have a function in neuronal differentiation and/or axon growth (Ring et al., 2015).

The epigenomic RDA, using lake type and lake system as explanatory variables, explained 3% (adjusted *R* ^2^ = 0.03) of the total variance (*F*_2,108_ = 2.5 *p*‐value = 0.001), of which lake system separated on the first axis (65%) and lake type on the second (35%) (Figure 5c). We identified 1493 loci clearly separating source and refuge populations (Figure 5d), of which 486 were found in or near genes. The GO analysis of these 486 loci recovered eighteen GO terms as significantly enriched (FDR < 0.1) and included nervous system development (e.g. GO:0007399, nervous system development; GO:0048699, generations of neurons; GO:0007409, axonogenesis; GO:0061564, axon development), cellular process (GO:0045595, regulation of cell differentiation; GO:0007154, cell communication) and developmental process (GO:0048856, anatomical structure development; GO:0050793; regulation of developmental process) (Table S11). The RDA that included fish age, in addition to lake type and lake system, continued to explain 3% of the total variance. Lake system and lake type were still resolved on axes 1 and 2, explaining 52% and 28% of the variance, respectively, while age on axis 3 explained 20% of the variance (Figure S8). These results indicated that lake type was a more important source of epigenomic variation than fish age, which we used here as a proxy for methylation that is associated with organismal growth, development and experience.

### 3.8 Genomic and epigenomic associations with eco‐morphology

**FIGURE 6:**
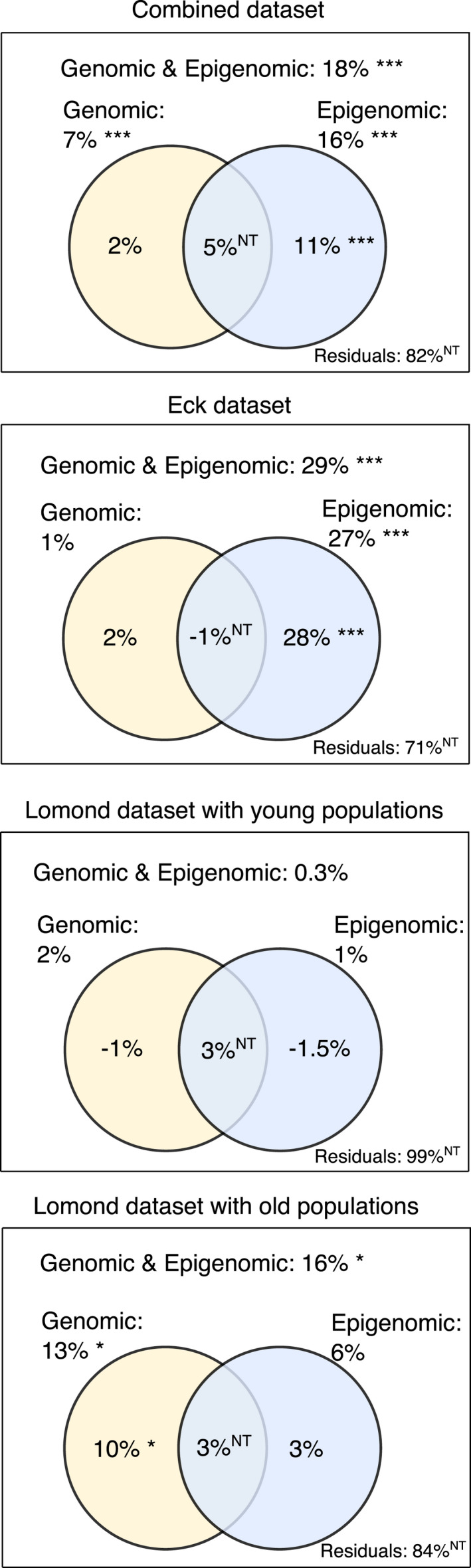
Proportion of morphological variation explained by genomic and epigenomic variation calculated using RDAs. Each panel of the variation partitioning decomposes the morphological variance in the combined, Eck, Lomond and young populations (7–9 years since translocation) data sets, and Lomond and old populations (30 year since translocation) data set. The total amount of morphological variance explained by the data corresponds to the ‘Genomic & Epigenomic’ category, while the remaining part is associated with the statistically nontestable (NT) ‘Residuals’. The proportion of variance associated with ‘Genomic’ (white), ‘Epigenomic’ (light blue) variation and their intersection (middle) are decomposed in the Venn diagrams. Significant effects are noted by the *

We applied RDA to partition the variance in morphology that was explained by genomic and epigenomic components. When considering the full data set, genomic and epigenomic effects together explained 18% (adjusted *R* ^2^ = 0.18) of the variance in body shape (*F*_6,90_ = 4.5, *p*‐value = 0.001). Genomic and epigenomic components separately explained 7% (adjusted *R* ^2^ = 0.07, *F*_3,93_ = 3.6, *p*‐value = 0.002) and 16% (adjusted *R* ^2^ = 0.16, *F*_3,93_ = 7.02, *p*‐value = 0.001) of the variance in morphology, respectively (Figure 6). However, when controlling for epigenomic effects, genomic effects did not explain any variation in morphology (adjusted *R* ^2^ = 0.02, *F*_3,90_ = 1.9, *p*‐value > 0.05), while when controlling for genomic variation, epigenomic variation explained 11% (adjusted *R* ^2^ = 0.11, *F*_3,90_ = 5.01, *p*‐value = 0.001) of the morphological variance. This pattern differed between systems and refuge population ages (Figure 6) and suggests that genomic effects may become more relevant for a population with time. In the Eck system analysis, genomic and epigenomic effects together explained 29% (adjusted *R* ^2^ = 0.29, *F*_6,33_ = 3.7, *p*‐value = 0.001) of the variance in body shape, genomic variation explained none (either alone or when controlling for epigenomic variation; *p*‐values > 0.1), while epigenomic variation explained 27% (adjusted *R* ^2^ = 0.29, *F*_3,36_ = 5.9, *p*‐value = 0.001) and 28% (adjusted *R* ^2^ = 0.28, *F*_3,33_ = 5.8, *p*‐value = 0.001) of the variation separately and when controlling for genomic variation, respectively (Figure 6). For the Lomond system, morphological variance in Lomond and the younger, that is 7‐ to 9‐year‐old refuge populations, was not explained by either genomic or epigenomic effects (all *p*‐values > 0.1). In the group containing Lomond and the older, that is 30‐year‐old refuge populations, genomic and epigenomic together explained 16% (adjusted *R* ^2^ = 0.16, *F*_6,30_ = 2.1, *p*‐value = 0.015) of the variance in morphology, genomic alone and when accounting for epigenomic effects explained 13% (adjusted *R* ^2^ = 0.13, *F*_3,33_ = 2.8, *p*‐value = 0.013) and 10% (adjusted *R* ^2^ = 0.10, *F*_3,30_ = 2.2, *p*‐value = 0.038), respectively, and epigenomic variation explained none (*p*‐values > 0.1).

## 4 DISCUSSION

By using a robust natural experiment involving multiple human‐mediated, purposeful conservation translocations, we found significant changes in populations of European whitefish for eco‐morphology, epigenomic and genomic patterns shortly following translocation. This represents only 2–10 generations (as age at fertility [Brown & Scott, 1994]). We found evidence of convergent morphology and similar extents of change among refuge populations regardless of time since translocation. Coupled with genomic evidence of differential selection pressures on the refuge populations at key genomic regions, we suggest this reflects consistent and rapid response to the shared environmental conditions in the translocation habitats. We identified common DNA methylation responses in refuge populations within and between translocation systems. Finally, we found a stronger correlation between morphological variation and epigenomic variation in the younger, that is 7‐ to 9‐year‐old translocated populations (Eck system), but a stronger correlation between morphological variation and genomic variation in the older, that is 30‐year‐old translocated populations (Lomond system). This suggests that the evolutionary responses to a novel environment for conservation translocations in early stages of the establishment (a few generations) may be mediated through plasticity and epigenomic effects but that in (slightly) more established translocations (ca. 10 generations), genomic changes become established.

### 4.1 Ecological consequences of translocation

We observed significant changes in morphology between all refuge populations compared with the source. As shown by the phenotypic trajectory analysis, these changes occurred in a convergent fashion and with similar magnitude, from notably different phenotypes in the source populations of Eck and Lomond to quite similar body shape in the refuge populations across systems. The observed differences in morphology likely have important consequences for the ecology of these populations (Siwertsson et al., 2013). While whitefish in Lomond and Eck have been shown to feed predominantly on pelagic (zooplankton) and benthic (macroinvertebrate) prey, respectively, and display morphological differences typical of variation associated with their respective diets (Etheridge et al., 2012), the refuge populations show intermediate phenotypes. This could be the result of a switch in the prey type utilized, due to the adoption of a more generalist diet or due to differences in the invertebrate communities between source and refuge lakes. The refuge lakes also differ from source lakes in surface area, depth and fish communities (Lyle et al., 2017), which have been shown to influence fish morphology (Kahilainen & Østbye, 2006; Recknagel et al., 2017; Siwertsson et al., 2013), and might explain some of the observed changes in body shape.

While populations of freshwater fishes are known to vary considerably in their morphology associated with the local environment and lake bathymetry (Jacobs et al., 2020; Recknagel et al., 2017; Siwertsson et al., 2013), it is striking that eco‐morphology in the novel environments results in similar patterns across translocated populations of whitefish regardless of lake of origin or time since colonization. Morphological divergence between translocated and source populations has been observed frequently among fish populations (Black et al., 2017; Collyer et al., 2007; Michaud et al., 2008). This effect is probably due to both phenotypic plasticity and local adaptation in response to biotic and abiotic differences between source and refuge environments. Previous research showed that fry from the source population Loch Lomond and refuge population of Loch Sloy raised in a common garden show similar phenotypic differences as those observed in wild, adult individuals (Koene et al., 2019), demonstrating that the phenotypic changes observed in the refuge populations do have a genetic component.

Different environments resulting in a change in diet between source and refuge populations are also suggested by both stomach content (short‐term/diet) and muscle (long‐term) stable isotope analysis. Refuge populations had the highest isotopic richness and with a greater inter‐individual range in δ^13^C, indicating a wide trophic niche width (Bearhop et al., 2004), a signal typical of more littoral feeding consumers compared with pelagic ones (France, 1995). In contrast, the source population had low isotopic richness (IRic) and isotopic diversity (IDiv), indicating a narrower foraging niche width (Cucherousset & Villegér, 2015). Furthermore, fish from the source population had high δ^15^N isotopes values compared with refuge populations, suggesting a higher trophic position and feeding more on pelagic food sources such as zooplankton (Syväranta and Jones, 2008), as observed before for these populations (Pomeroy, 1991). The lower δ^15^N in the refuge populations suggests a diet dominated by littoral macroinvertebrates (Syväranta and Jones, 2008). Because the refuge lakes possess a much reduced fish community compared to the source lakes (Lyle et al., 2017), the expansion in niche width and diet switch observed in the refuge populations may be the result of ecological release (Bolnick et al., 2010), when a colonizing species can expand its trophic niche by utilizing new resources that may have been taken by competitors in the original environment. Consequently, our findings show that rapid ecological release may be an important component of conservation management by translocation.

Whitefish are renowned for high levels of polymorphism and adaptive eco‐morphologies in body shape, gill rakers and physiology (Evans et al., 2013; Jacobs et al., 2019; Kahilainen & Østbye, 2006; Laporte et al., 2016; Siwertsson et al., 2013). The genetic similarity and shared evolutionary history of the European whitefish species complex at large geographic areas (Rougeux, Gagnaire, & Bernatchez, 2019; Rougeux, Gagnaire, Præbel, et al., 2019), despite high levels of local variation at the smaller scale (Doenz et al., 2018; Rougeux et al., 2017), could suggest the same mechanisms underlie our findings on rapid divergence following translocation. In fact, genetic and epigenetic foundations for these eco‐morpho‐physiological traits and their rapid evolution have been shown in other whitefish systems (Jacobs et al., 2019; Laporte et al., 2015, 2019).

### 4.2 Population genomic consequences of translocation

The observed levels of reduced genetic diversity in all the refuge populations, and their increased inbreeding and relatedness, suggest the population genomic consequences of these translocations are predictable. The diversity decline was more evident in the 30‐year‐old refuge populations of whitefish, which were established with a much smaller number of families and fewer released individuals, which we suggest meant less starting genetic variation and a stronger bottleneck. In addition, even among the 7‐ to 9‐year‐old refuge populations we found an effect of founding group size. For example, refuge populations from the Eck system had less reduction in heterozygosity and inbreeding compared with those of the Lomond system, and Eck system was established with larger founding group size. Our findings indicate that founder size is an important factor when planning conservation translocations (Allendorf & Lundquist, 2003; Szűcs et al., 2017).

Furthermore, we observed low but significant genetic differentiation between source and refuge populations, with *F*_ST_ in the 30‐year‐old translocated populations being an order of magnitude higher compared with the 7–9 year‐old ones. This also could be due to different founder size and also longer time of divergence and genetic drift (Groombridge et al., 2012; Szűcs et al., 2017). However, because we are exploring previous human‐induced changes in a limited number of populations, it is not possible to tease apart those influences. These reductions in genetic diversity and heterozygosity, and increases in inbreeding, in the refuge whitefish populations are not extreme, but an assessment of their stability over time and if there is an effect on fitness would be valuable. Genetic monitoring of the refuge populations is needed out at regular intervals to detect possible genetic diversity loss over time and consider mitigation measures (Schwartz et al., 2007).

### 4.3 Consistent signals of local adaptation in refuge populations

The absence of gene flow and presence of selective pressures due to environmental differences can push the evolution of source and refuge populations on separate trajectories (Vincent et al., 2013). Thus, the use of multiple, independent translocations in this study is a powerful way to gain insights into the process of rapid adaptation to the local environment and to identify the functional regions under selection during initial population divergence. Across lake systems, we found five genes putatively under differential selection in refuge populations compared with source and that might be involved in local adaptation to the new environments. Two of these genes, ladderlectin and TLR3, are involved in the immune system. TLR3 is an immune receptor specialized in recognizing double‐stranded RNA viruses (Sahoo et al., 2015), while ladderlectin is a protein involved in pathogen elimination with the ability to bind Gram‐negative bacteria and chitin (Russell et al., 2008). In addition, DnaJC18, which is part of the Heat shock protein (HSP) family Hsp40, has been found to be upregulated following bacterial infection in catfish (Song et al., 2014), indicating a role in immune system response. Local adaptation in the immune system in response to novel environmental conditions and habitat‐specific parasite communities is abundant in freshwater fish (Eizaguirre et al., 2012; Pavey et al., 2013) and consistent with earlier work showing differences in parasite load and infection rate in source compared with the two 30‐year‐old refuge populations of whitefish (Etheridge et al., 2010). Because we found these genes across all refuge populations, it suggests an aspect of identical molecular responses to selection pressures in the immune response. Other relevant candidate genes included involvement in nervous system development, such as GPRIN3, a gene active in the physiology of the striatum, a part of the brain involved in the motor system (Karadurmus et al., 2019), and Atp8b1, a lipid metabolism transport gene whose mutations are associated with cholestasis liver disease (Pham et al., 2017). Overall, genomic outliers shared by refuge populations suggest immune response, nervous system and metabolism functions are among the first to be impacted and strongly under selection when fishes colonize and adapt to new environments (Elmer et al., 2010; Marques et al., 2018; Terekhanova et al., 2014; Vatsiou et al., 2016). This may reflect new evolutionary trajectories in translocated populations.

### 4.4 Epigenomic consequences of translocation

Epigenetic mechanisms, such as DNA methylation, provide a molecular route to phenotypic plasticity (Angers et al., 2020), which plays a central role in facilitating the establishment and persistence of populations in new habitats (Lande, 2015). We detected shared differentially methylated loci between source and refuge populations across lake systems, reflecting consistent response by the epigenome to translocation. Several differentially methylated loci were in or near genes involved in neural functions. For example, DPYSL5 is involved in neural development and research showed it had reduced expression in rainbow trout (*Oncorhynchus mykiss*) offspring from thermally stressed mothers, with their fear‐related locomotor response and spatial learning abilities impaired (Colson et al., 2019). Candidate gene ZNF367 is a core regulating gene during brain development in teleost fish (Baumgart et al., 2014). Another identified locus was near the gene SYN3, which plays an important role in early neural differentiation and in neuronal progenitor cell development (Garbarino et al., 2014), and in a salmonid‐wide analysis was found to be under intensified and diversifying selection in the genus *Coregonus* (Schneider et al., 2019). However, caution is needed in interpreting any functional role of the differentially methylated loci because of the reduced representation approach we applied and because we examined terminal tissues with unknown link between methylation and developmental consequences.

DNA methylation is a complex mechanism with most influence being due to the role of differentially methylated regions rather than single loci, as most epigenetic variants are not deterministic epi‐alleles with defined location and effects but interactive regulatory factors (Adrian‐Kalchhauser et al., 2020; van der Graaf et al., 2015). Rather than aiming to definitively identify functional molecular consequences of methylation, the motivation of our experiment was to infer whether there is population‐level signal of epigenomic response to translocation and whether it holds ecologically and evolutionarily valuable signal, thereby prompting future research to examine in more molecular and developmental detail. Indeed, we showed that conservation translocations lead to significant changes in patterns of DNA methylation. Our results are consistent with a potential role of epigenomic variation in adaptation to novel environments that warrants further study. Future high‐density genome‐wide research of methylation would be valuable for inferring functional targets and responses across translocation environments.

This effect is evident in our finding that lake type (i.e. being refuge or source) explained substantially more variance in the epigenomic models than it did in the genomic models (35% vs 3% of the variance captured in the RDA). This agrees with previous findings that individuals reared in different environments exhibit higher epigenomic differentiation than genomic differentiation due to phenotypic plasticity (Artemov et al., 2017; Le Luyer et al., 2017; Gavery et al., 2018), suggesting a rapid and strong effect of environments on DNA methylation. Furthermore, we found some evidence that epigenomic variation had stronger association with morphological variation in younger, less genetically differentiated refuge populations (35%), while genomic variation had stronger association with morphological variance in the older, more genetically differentiated refuge populations (16%). However, the morphological variation explained by either genomic or epigenomic variation was low in all comparisons, consistent with a previous study examining the influence of these two factors on gene expression (Rougeux, Laporte, Gagnaire, & Bernatchez, 2019). This suggests that phenotypic changes in translocated populations might be influenced by other factors that remain to be evaluated and warrant further exploration.

## 5 CONCLUSIONS

We identified consistent ecological and morphological responses in whitefish refuge populations, suggesting that the ecological and evolutionary consequences of these conservation‐driven translocations might be predictable. In the refuge populations, genetic diversity was reduced while relatedness and inbreeding increased; this was related to both the number of founder individuals and time since translocation and are difficult to separate. Genomic and epigenomic analyses suggested roles of neural development, immune system and metabolism in response to translocation. This demonstrates that transgenerational molecular mechanisms might facilitate acclimation and rapid adaptation to new environments and response to divergent selection that accompany these human‐mediated colonization. In addition, our findings suggest that translocated populations can adapt to their new environments at the genomic level despite a reduction in diversity. Our findings shed light on processes behind recent and rapid differentiation, acclimation and adaptation in populations of high conservation concern and that are targets of management effort. This highlights the value of combining genomic and epigenomic approaches to understand ecological and evolutionary responses to novel environments, but also the need for experimental work to better understand the role, and potential transgenerational effect, of epigenomic mechanisms.

## CONFLICT OF INTEREST

None declared.

6

## Supporting information

Fig S1‐8

Table S1‐11

## Data Availability

The data that support the findings of this study, and the scripts used, are openly available in University of Glasgow Enlighten Repository [https://doi.org/10.5525/gla.researchdata.1078]. Short read data are available at NCBI SRA [PRJNA658243].
