# Supplementary material for: Rapid adaptation through genomic and epigenomic responses following translocations in an endangered salmonid
Source: Evol Appl. 2021 Jul 6;14(10):2470–89. doi: 10.1111/eva.13267 (PMC8549615; doi:10.1111/eva.13267)
Supplement: Supplementary file 1 — Fig S1‐8 [file EVA-14-2470-s002.docx]

**Supplementary figures**

**Rapid adaptation through genomic and epigenomic mechanisms following translocations in an endangered salmonid**

Crotti, M.^1^, Yohannes, E.^3^, Winfield, I.J.^4^, Lyle, A.A.^2^, Adams, C.E.^1,2^, Elmer, K.R.^1^

Figure S1. Landmarks and linear traits used for the morphological analyses. a) The 14 landmarks used for the geometric morphometric analysis. b) Linear traits obtained by measuring distance between landmarks. ML = maxilla length, SL = snout length, HD = head depth, ED = eye diameter, HL = head length, FL = fin length, BDA = body depth anterior, BDP = body depth posterior, CPD = caudal peduncle depth.

Figure S2. Morphological and stable isotope analyses. a) Principal component analysis for all populations. Large dots represent mean score for each population while small dots are individuals. Ellipses represent 50% confidence intervals. b) Phenotypic trajectory analysis for source and refuge populations across lake systems. c) Relationship between δ^15^N from muscle and stomach content data.

Figure S3. Results of the linear traits analysis. Statistical results are reported in Table S2.

Figure S4. Impact of founder size on genetic diversity, inbreeding, and relatedness. Y axis represents the difference in average estimate between source and refuge populations. X axis is the number of families used to create the refuge populations (data from Lyle et al., 2017).

Figure S5. Results of the *Admixture* analysis for the genomic Lomond and Eck system datasets separately. For the Lomond system, K = 2 was best fitting number of clusters, separating the refuge population of Sloy from Lomond, followed by K = 3, additionally separating the Carron population. For the Eck system, K = 1 was most likely scenario, with no refuge population being clearly separated from the source population.

Figure S6. Principal component analyses of the epigenomic combined dataset, showing PC1 and PC2 (a), PC3 and PC4 (b).

Figure S7. Distribution of all loci and DM loci in the epigenomic combined, epigenomic Eck, and epigenomic Lomond datasets across genomic regions.

Figure S8. Epigenomic RDA with age, lake type, and lake system as explanatory variables. Lake system and lake type were displayed on axis 1 and 2 (a), while age displayed on axis 3 (b).
